# Secrecy Performance of TAS/SC-Based Multi-Hop Harvest-to-Transmit Cognitive WSNs Under Joint Constraint of Interference and Hardware Imperfection

**DOI:** 10.3390/s19051160

**Published:** 2019-03-07

**Authors:** Phu Tran Tin, Pham Minh Nam, Tran Trung Duy, Phuong T. Tran, Miroslav Voznak

**Affiliations:** 1 VSB, Technical University of Ostrava, 17. listopadu 15/2172, 708 33 Ostrava, Poruba, Czech Republic; phutrantin@iuh.edu.vn (P.T.T.); miroslav.voznak@vsb.cz (M.V.); 2 Faculty of Electronics Technology, Industrial University of Ho Chi Minh City, Ho Chi Minh City 700000, Vietnam; 1727002@student.hcmute.edu.vn; 3 Faculty of Electrical and Electronics Engineering, HCMC University of Technology and Education, Ho Chi Minh City 700000, Vietnam; 4 Department of Telecommunications, Posts and Telecommunications Institute of Technology, Ho Chi Minh City 700000, Vietnam; trantrungduy@ptithcm.edu.vn; 5 Wireless Communications Research Group, Faculty of Electrical and Electronics Engineering, Ton Duc Thang University, Ho Chi Minh City 700000, Vietnam

**Keywords:** multi-hop wireless sensor networks, physical-layer security, transmit antenna selection, selection combining, cognitive radio, energy harvesting, hardware impairments

## Abstract

In this paper, we evaluate the secrecy performance of multi-hop cognitive wireless sensor networks (WSNs). In the secondary network, a source transmits its data to a destination via the multi-hop relaying model using the transmit antenna selection (TAS)/selection combining (SC) technique at each hop, in the presence of an eavesdropper who wants to receive the data illegally. The secondary transmitters, including the source and intermediate relays, have to harvest energy from radio-frequency signals of a power beacon for transmitting the source data. Moreover, their transmit power must be adjusted to satisfy the quality of service (QoS) of the primary network. Under the joint impact of hardware imperfection and interference constraint, expressions for the transmit power for the secondary transmitters are derived. We also derive exact and asymptotic expressions of secrecy outage probability (SOP) and probability of non-zero secrecy capacity (PNSC) for the proposed protocol over Rayleigh fading channel. The derivations are then verified by Monte Carlo simulations.

## 1. Introduction

Security is one of the important issues in wireless sensor networks (WSNs) due to the broadcast nature of wireless medium. Conventionally, encryption/decryption algorithms that generate public/private keys are used to guarantee security [1,2]. Recently, a security framework for the physical layer, called the wiretap channel or physical-layer security (PLS) [3,4,5,6,7], has been introduced as a potential solution. In PLS, the difference between the channel capacity of the data link and the channel capacity of the eavesdropping link, named secrecy capacity, is commonly used to evaluate secrecy performance such as average secrecy capacity (ASC), secrecy outage probability (SOP), and probability of non-zero secrecy capacity (PNSC). Hence, to enhance the secrecy performance, the quality of the data and eavesdropping links should be increased and decreased, respectively. To enhance the channel capacity of the data links, diversity transmit and receive methods [8,9,10] can be used. In [8], the transmit antenna selection (TAS) technique is employed at a multi-antenna base station (BS) to maximize the instantaneous signal-to-noise ratios (SNRs) obtained between BS and intended users. References [9,10] considered MIMO secure communication systems, where the transmitter uses the TAS technique, while the legitimate receiver and eavesdropper can use selection combining (SC) or maximal ratio combining (MRC) for reception. In [11,12,13,14,15], cooperative relaying strategies were proposed to improve secrecy performance via increasing the channel capacity for the data links. Moreover, to avoid the eavesdropper combining the data with MRC, a randomize-and-forward (RF) strategy [16] can be employed, where the source and relays generate different code-books to confuse the eavesdropper. For significantly degrading the eavesdropping channels, cooperative jamming (CJ) methods were reported in [17,18,19]. The basic idea of the CJ method is that jammer nodes are employed to generate artificial noises on the eavesdropper. In addition, the legitimate receivers have to cooperate with the jammers to remove the interference appeared in their received signals. However, the implementation of this technique can be a difficult work due to the requirement of high synchronization between the nodes.

Recently, wireless energy harvesting (EH) [20,21] has emerged as a potential solution to prolong the lifetime of WSNs. In wireless EH, the energy-constrained devices can harvest energy from radio frequency signals generated by ambient nodes. In [21], EH amplify-and-forward (AF) relaying protocols were proposed and analyzed, where the relay node harvests energy from the source for transmitting the source data to the destination. The authors of [22] considered both AF and decode-and-forward (DF) relaying schemes employed a hybrid power splitting (PS) and time switching (TS) EH relay. In [23,24,25], power beacons (PBs) are deployed in the networks to charge energy for wireless devices. The PB-aided wireless power transfer models are suitable for large-scale WSNs or wireless ad-hoc networks. Moreover, to avoid causing the co-channel interference to the receivers, channels used for harvesting energy from the wireless signals of PBs are different from those used for the data transmission. In [23,24,25], the authors studied the performance of secondary networks in PB-assisted underlay cognitive radio (PB-UCR), where the transmit power of secondary users was limited by both the harvested energy and the maximum interference levels required by primary users. In [26], a secure communication scenario in cognitive sensor radio networks with an EH eavesdropper was introduced. Reference [27] proposed various path-selection protocols in multi-path multi-hop relaying networks in the presence of active eavesdroppers. Moreover, in [27], all of the terminals whose transceiver hardware is low-cost suffered from hardware imperfection due to phase noise, I/Q imbalance (IQI), amplifier nonlinearities, etc. [28,29,30].

This paper deals with multi-hop secure communication networks in PB-UCR under impact of hardware impairments. In [31], the authors first evaluated the secrecy capacity in the presence of IQI for OFDMA communication systems. Reference [32] proposed a secure massive MIMO system with a passive multiple-antenna eavesdropper and hardware noises. The results in [31,32] showed that the hardware impairments significantly impact of the secrecy performance. Next, unlike [27], our scheme considers the PB-URC networks using the MIMO-based TAS/SC relaying technique. In [33,34,35], cooperative multi-hop full-duplex relaying networks were proposed to enhance the end-to-end secrecy performance. However, it is too difficult to apply these schemes into WSNs due to the complexity of full-duplex operation. References [36,37,38] introduced simple multi-hop secure relaying scenarios in which all of the nodes are equipped with a single antenna. In [39], PLS in downlink MIMO multi-hop heterogeneous cellular networks were investigated. In particular, the data transmission between base stations and mobile users is realized via direct or multi-hop mode. In [40], a multi-hop multicasting secure transmission protocol with multi-antenna DF relays in the presence of multiple eavesdroppers was proposed and analyzed. However, in [36,37,38,39,40], the authors did not consider the cognitive environment as well as the wireless EH technique.

To the best of our knowledge, there is no published work related to multi-hop secure transmission in PB-UCR under the impact of hardware noises. In the proposed protocol, a secondary source transmits its data to a secondary destination using the multi-hop mode in the presence of a secondary eavesdropper. To support the reliable communication at each hop, the TAS/SC technique is used to forward the source data. Operating on the underlay spectrum sharing method, the transmit power of the secondary source and relay nodes must be adjusted to satisfy the required QoS of the primary network. Moreover, the secondary transmitters have to harvest energy from PB deployed in the secondary network for the data transmission. The main contribution of this paper can be summarized as follows:

- We propose a simple multi-hop MIMO relaying protocol using the TAS/SC technique for PB-UCR WSNs. The proposed protocol can obtain energy efficiency and spectrum usage efficiency, enhance the reliability of data transmission, and improve the secrecy performance.
- In almost published works related to EH and UCR (see [23,24,25]), the transmitters adjust their transmit power following the instantaneous channel state information. As a result, the transmit power is a random variable (RV), which is not feasible. In this paper, the secondary source and relay nodes are assumed to transmit the source data at fixed transmit power levels. In addition, we derive an exact closed-form expression of the average transmit power of the secondary transmitters under the joint impacts of the energy harvested, the interference constraint, and the maximum transmit power level.
- We investigate the impact of hardware impairments on the end-to-end SOP and PNSC of the proposed scheme. Indeed, the obtained results presented that the hardware imperfection has a significant impact on the secrecy performance. Moreover, the analytical results showed that different values of the hardware impairment levels of the data and eavesdropping links lead to different secrecy performance trend. Finally, it is worth noting that the proposed scheme is a generalized case of the existing schemes in which the transceiver hardware is assumed to be perfect [11,41,42,43].
- We derive new exact and asymptotic expressions of the end-to-end SOP and PNSC over Rayleigh fading channels, which are then verified by Monte Carlo simulations.

The rest of this paper is organized as follows. The system model of the considered protocol is described in Section 2. In Section 3, the expressions of SOP are derived. The simulation results are shown in Section 4. Finally, this paper is concluded in Section 5.

## 2. System Model

**Figure 1:**
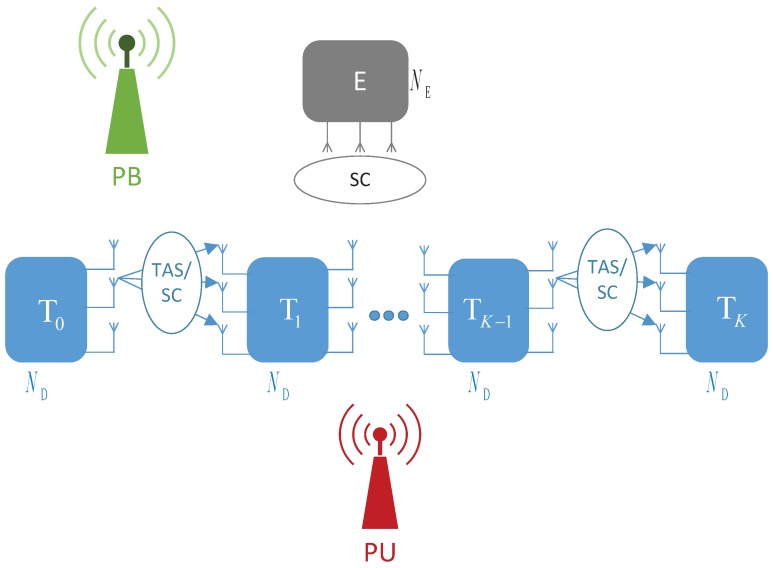
System model of the proposed scheme.

Figure 1 illustrates the system model of the proposed scheme, in which a secondary source $\left(T_{0}\right)$ wants to transmit its data to a destination $\left(T_{K}\right)$ via K−1 intermediate nodes denoted by $T_{1},$$T_{2}$, …, $T_{K-1}$. The data transmission between $T_{0}$ and $T_{K}$ is realized via *K* orthogonal time slots, exploiting the TAS/SC technique. In particular, at the $\left(k+1\right)$-th hop, the node $T_{k}$ selects an antenna to transmit the source data to the node $T_{k+1}$, which uses the SC technique to combine the signals received from $T_{k}$, where k=0,1,…,K. We assume that all of the nodes including the source, the destination and the relays are equipped with $N_{D}$ antennas. The transmitter $T_{k}$ has to harvest energy from wireless signals of a single-antenna power beacon (PB) deployed in the secondary network. Moreover, $T_{k}$ must adjust the transmit power to satisfy a maximal interference threshold ($I_{\mathrm{th}}$) required by a single-antenna primary user (PU). Also in the secondary network, there exists an $N_{E}$-antenna eavesdropper (E) who uses the SC technique to decode the source data obtained at each hop. Similar to [16], the secondary transmitters randomly generate code-book to confuse the eavesdropper.

**Comment 1**: Due to the size limitation and complexity constraint, it is assumed that all of the receivers can only use the SC combiner for decoding the data. In addition, the secrecy performance of the proposed protocol is same with that of the corresponding one with multiple non-colluding single-antenna eavesdroppers [44,45]. Finally, in the case where the eavesdropper can employ the MRC technique, the expressions derived in this paper can be used as bound expressions of the secrecy performance.

Let us denote $\gamma_{X,Y}$ as the channel gain of the link, where $X,Y\in \left\{T_{k},E,\mathrm{PB},\mathrm{PU}\right\}$. Assume that all of the channels are Rayleigh fading; hence the channel gain $\gamma_{X,Y}$ is an exponential RV. The cumulative distribution function (CDF) and probability density function (PDF) of $\gamma_{X,Y}$ can be expressed, respectively, as

(1) $$\begin{array}{c} F_{\gamma_{X,Y}}\left(u\right)=1-\exp\left(-\lambda_{X,Y}u\right),\;\;f_{\gamma_{X,Y}}\left(u\right)=\lambda_{X,Y}\exp\left(-\lambda_{X,Y}u\right), \end{array}$$

where $\lambda_{X,Y}$ is parameter of the exponential RV $\gamma_{X,Y}$, i.e., $\lambda_{X,Y}=1/E\left\{\gamma_{X,Y}\right\}=1/\sqrt{Var\left\{\gamma_{X,Y}\right\}}$, where E is expected operator and $Var\left\{X\right\}$ is variance of X. To take path-loss into account, we can model $\lambda_{X,Y}$ as $\lambda_{X,Y}=d_{X,Y}^{\beta }$ [46], where $d_{X,Y}$ is the distance between X and Y, and β is the path-loss exponent.

We denote $\gamma_{T_{k,m},Y}$ as the channel gain between the *m*-th antenna of $T_{k}$ and Y, where $m=1,2,\ldots ,N_{D}$. Assume that RVs $\gamma_{T_{k,m},Y}$ are independent and identical, i.e., $\lambda_{T_{k,m},Y}=\lambda_{T_{k},Y}$ for all *m*. Also, let $\gamma_{X,T_{k+1,n}}$ and $\gamma_{X,E_{p}}$ as channel gain between X and the *n*-th antenna of $T_{k+1}$, and that between X and the *p*-th antenna of E, respectively, where $n=1,2,\ldots ,N_{D}$ and $p=1,2,\ldots ,N_{E}$. Similarly, $\gamma_{X,T_{k+1,n}}$ and $\gamma_{X,E_{p}}$ are independent and identically distributed (i.i.d.) RVs, i.e., $\lambda_{X,T_{k+1,n}}=\lambda_{X,T_{k+1}}$ and $\lambda_{X,E_{p}}=\lambda_{X,E}$ for all *n* and *p*.

Let T denote the total transmission time between $T_{0}$ and $T_{K}$, and hence the time allocated for each time slot is given as τ=T/K. Considering the $\left(k+1\right)$-th hop; a duration of ετ is used for $T_{k}$ to harvest energy, and the remaining duration, i.e., $\left(1-\varepsilon \right)\tau$, is spent for the data transmission between $T_{k}$ and $T_{k+1}$, where 0≤ε≤1. Then, the amount of energy that $T_{K}$ can harvest is given by

(2) $$\begin{array}{c} EH_{k}=\eta \varepsilon \tau P_{B}\sum_{v=1}^{N_{D}}\gamma_{\mathrm{PB},T_{k,v}}, \end{array}$$

where $\eta \left(0\leq \eta \leq 1\right)$ is energy conversion efficiency, and $P_{B}$ is transmit power of PB.

From (2), we can formulate the transmit power that $T_{k}$ can use in the data transmission phase by

(3) $$\begin{array}{c} Q_{\mathrm{EH},k}=\frac{EH_{k}}{\left(1-\varepsilon \right)\tau }=\chi P_{B}\varphi_{\mathrm{sum},k}, \end{array}$$

where $\chi =\eta \varepsilon /\left(1-\varepsilon \right)$ and $\varphi_{\mathrm{sum},k}=\sum_{v=1}^{N_{D}}\gamma_{\mathrm{PB},T_{k,v}}$. Using (Equation (A.7) [25]), we can write the CDF of $\varphi_{\mathrm{sum},k}$ as

(4) $$\begin{array}{c} F_{\varphi_{\mathrm{sum},k}}\left(z\right)=1-\sum_{t=0}^{N_{D}-1}\frac{1}{t!}{\left(\lambda_{\mathrm{PB},T_{k}}z\right)}^{t}\exp\left(-\lambda_{\mathrm{PB},T_{k}}z\right). \end{array}$$

Considering the data transmission between the transmitter A and the receiver B; under the impact of the hardware impairments, the instantaneous SNR received at B can be expressed as in [30,33,34,47,48]:

(5) $$\begin{array}{c} \Psi_{A,B}=\frac{P_{A}\gamma_{A,B}}{\left(\kappa_{A}^{2}+\kappa_{B}^{2}\right)P_{A}\gamma_{A,B}+\sigma^{2}}=\frac{P_{A}\gamma_{A,B}}{\kappa_{A,B}^{2}P_{A}\gamma_{A,B}+\sigma^{2}}, \end{array}$$

where $A,B\in \left\{T,E,\mathrm{PB},\mathrm{PU}\right\}$, $P_{A}$ is transmit power of the transmitter A, $\gamma_{A,B}$ is channel gain between A and B, $\sigma^{2}$ is the variance of additive white Gaussian noise (AWGN), $\kappa_{A}^{2}$ and $\kappa_{B}^{2}$ are constants characterizing the level of the hardware impairments at A and B, respectively, and $\kappa_{A,B}^{2}=\kappa_{A}^{2}+\kappa_{B}^{2}$ is the total hardware impairment level. The values of $\kappa_{A}^{2}$, $\kappa_{B}^{2}$, $\kappa_{A,B}^{2}$, which depend on the structure of the transceiver hardware at A and B, can be determined by practical experiments.

Moreover, in the URC network, if $T_{k}$ uses the *m*-th antenna to transmit the data, the transmit power must satisfy the interference constraint required by PU as in [25,49]:

(6) $$\begin{array}{c} Q_{\mathrm{IN},k,m}=\frac{I_{\mathrm{th}}}{\left(1+\kappa_{P}^{2}\right)\gamma_{T_{k,m},\mathrm{PU}}}, \end{array}$$

where $\kappa_{P}^{2}$ is total hardware impairment level at $T_{k}$ and PU (see [25,49]).

Let $P_{S}$ denote the maximum transmit power of each antenna; by combining (3) and (6), the transmit power of the *m*-th antenna at $T_{k}$ can be formulated by

(7) $$\begin{array}{cc} P_{k,m} & =\min\left(Q_{\mathrm{EH},k},Q_{\mathrm{IN},k,m},P_{S}\right)=P_{S}\min\left(\mu_{1}\varphi_{\mathrm{sum},k},\frac{\mu_{2}}{\gamma_{T_{k,m},\mathrm{PU}}},1\right)\\ & =\left\{\begin{array}{c} P_{S}\min\left(\mu_{1}\varphi_{\mathrm{sum},k},\frac{\mu_{2}}{\gamma_{T_{k,m},\mathrm{PU}}}\right),\;\;\;\mathrm{if}\;\min\left(\mu_{1}\varphi_{\mathrm{sum},k},\frac{\mu_{2}}{\gamma_{T_{k,m},\mathrm{PU}}}\right)<1\\ P_{S},\;\;\;\;\;\;\;\;\;\;\;\;\;\;\;\;\;\;\;\;\;\;\;\;\;\;\;\;\;\;\;\;\;\;\;\;\;\;\;\;\;\;\;\;\;\;\;\;\;\;\;\;\;\;\;\;\;\;\;\;\;\;\;\;\;\;\;\;\;\mathrm{if}\;\min\left(\mu_{1}\varphi_{\mathrm{sum},k},\frac{\mu_{2}}{\gamma_{T_{k,m},\mathrm{PU}}}\right)\geq 1 \end{array}\right. \end{array}$$

where

$$\begin{array}{c} \mu_{1}=\frac{\chi P_{B}}{P_{S}},\;\;\mu_{2}=\frac{I_{\mathrm{th}}}{\left(1+\kappa_{P}^{2}\right)P_{S}}. \end{array}$$

We can observe from (7) that when $\min\left(\mu_{1}\varphi_{\mathrm{sum},k},\mu_{2}/\gamma_{T_{k,m},\mathrm{PU}}\right)<1$, then $P_{k,m}=P_{S}\min\left(\mu_{1}\varphi_{\mathrm{sum},k},\mu_{2}/\gamma_{T_{k,m},\mathrm{PU}}\right)$ is a RV, which is not feasible (although this assumption was widely used in many published literature, e.g., [23,24,25] and references therein). In practice, the transmit power $P_{k,m}$ should be fixed at pre-determined levels. Indeed, assume that there are W+1 fixed levels denoted by $L_{v}\left(v=0,1,\ldots ,W\right)$ with $L_{v}=vP_{S}/W$. For example, $L_{0}=0$ is the lowest level, and $L_{W}=P_{S}$ is the highest one. Now, by combining with (7), we can write the expression of $P_{k,m}$ as

(8) $$\begin{array}{c} P_{k,m}=\left\{\begin{array}{c} 0,\;\;\;\;\;\;\;\;\mathrm{if}\;\;P_{S}\min\left(\mu_{1}\varphi_{\mathrm{sum},k},\frac{\mu_{2}}{\gamma_{T_{k,m},\mathrm{PU}}}\right)<L_{1}\\ L_{v},\;\;\;\;\;\mathrm{if}\;\;v<W,\;\;L_{v}\leq P_{S}\min\left(\mu_{1}\varphi_{\mathrm{sum},k},\frac{\mu_{2}}{\gamma_{T_{k,m},\mathrm{PU}}}\right)<L_{v+1}\;\;\\ L_{W},\;\;\;\mathrm{if}\;L_{W}\leq P_{S}\min\left(\mu_{1}\varphi_{\mathrm{sum},k},\frac{\mu_{2}}{\gamma_{T_{k,m},\mathrm{PU}}}\right)\; \end{array}\right. \end{array}$$

Now, we consider the data transmission between $T_{k}$ and $T_{k+1}$. Employing the TAS/SC technique, the transmit antenna at $T_{k}$ and the receive antenna at $T_{k+1}$ are selected by the following strategy (see [9]):

(9) $$\begin{array}{c} \gamma_{T_{k,b},T_{k+1,c}}=\max_{m=1,2,\ldots ,N_{D}}\left(\max_{n=1,2,\ldots ,N_{D}}\left(\gamma_{T_{k,m},T_{k+1,n}}\right)\right), \end{array}$$

where *b* and *c* are the selected antennas at $T_{k}$ and $T_{k+1}$, respectively, $b\in \left\{1,2,\ldots ,N_{D}\right\}$ and $c\in \left\{1,2,\ldots ,N_{D}\right\}$. Since $\gamma_{T_{k,m},T_{k+1,n}}$ are i.i.d. RVs, CDF of $\gamma_{T_{k,b},T_{k+1,c}}$ can be obtained by

(10) $$\begin{array}{cc} F_{\gamma_{T_{k,b},T_{k+1,c}}}\left(x\right) & =\prod_{m=1}^{N_{D}}\prod_{n=1}^{N_{D}}F_{\gamma_{T_{k,m},T_{k+1,n}}}\left(x\right)={\left(1-\exp\left(-\lambda_{T_{k},T_{k+1}}x\right)\right)}^{N_{D}^{2}}\\ & =1+\sum_{v=1}^{N_{D}^{2}}{\left(-1\right)}^{v}\left(\begin{array}{c} N_{D}^{2}\\ v \end{array}\right)\exp\left(-v\lambda_{T_{k},T_{k+1}}x\right). \end{array}$$

With the presence of hardware impairments, the channel capacity of the $T_{k}\to T_{k+1}$ link is calculated as

(11) $$\begin{array}{c} C_{D,k}=\left(1-\varepsilon \right)\tau \log_{2}\left(1+\frac{P_{k,b}\gamma_{T_{k,b},T_{k+1,c}}}{\kappa_{D}^{2}P_{k,b}\gamma_{T_{k,b},T_{k+1,c}}+\sigma^{2}}\right), \end{array}$$

where $\kappa_{D}^{2}$ is the total hardware impairment level at $T_{k}$ and $T_{k+1}$, which is assumed to be the same for all values of *k*.

Let us consider the eavesdropping link at the $\left(k+1\right)$-th hop; the channel capacity of the $T_{k}\to E$ link can be obtained by

(12) $$\begin{array}{c} C_{E,k}=\left(1-\varepsilon \right)\tau \log_{2}\left(1+\frac{P_{k,b}\gamma_{T_{k,b},E_{g}}}{\kappa_{E}^{2}P_{k,b}\gamma_{T_{k,b},E_{g}}+\sigma^{2}}\right), \end{array}$$

where $\kappa_{E}^{2}$ is the total hardware impairment level at $T_{k}$ and E. In addition, since E uses the SC combiner, the channel gain $\gamma_{T_{k,b},E_{g}}$ can be written as

(13) $$\begin{array}{c} \gamma_{T_{k,b},E_{g}}=\max_{p=1,2,\ldots ,N_{E}}\left(\gamma_{T_{k,b},E_{p}}\right), \end{array}$$

where $g\in \left\{1,2,\ldots ,N_{E}\right\}$. In addition, CDF of $\gamma_{T_{k,b},E_{g}}$ can be expressed by

(14) $$\begin{array}{c} F_{\gamma_{T_{k,b},E_{g}}}\left(x\right)=\prod_{p=1}^{N_{E}}F_{\gamma_{T_{k,b},E_{p}}}\left(x\right)={\left(1-\exp\left(-\lambda_{T_{k},E}x\right)\right)}^{N_{E}}. \end{array}$$

From (14), we obtain PDF of $\gamma_{T_{k,b},E_{g}}$ as

(15) $$\begin{array}{cc} f_{\gamma_{T_{k,b},E_{g}}}\left(x\right) & =N_{E}\lambda_{T_{k},E}\exp\left(-\lambda_{T_{k},E}x\right){\left(1-\exp\left(-\lambda_{T_{k},E}x\right)\right)}^{N_{E}-1}\\ & =\sum_{u=0}^{N_{E}-1}{\left(-1\right)}^{u}\left(\begin{array}{c} N_{E}-1\\ u \end{array}\right)N_{E}\lambda_{T_{k},E}\exp\left(-\left(u+1\right)\lambda_{T_{k},E}x\right). \end{array}$$

Next, from (11) and (12), the secrecy capacity at the $\left(k+1\right)$-th hop is obtained by

(16) $$\begin{array}{c} C_{\mathrm{Sec},k}=\max\left(0,C_{D,k}-C_{E,k}\right)\\ =\max\left(0,\left(1-\alpha \right)\tau \left[\log_{2}\left(1+\frac{P_{k,b}\gamma_{T_{k,b},T_{k+1,c}}}{\kappa_{D}^{2}P_{k,b}\gamma_{T_{k,b},T_{k+1,c}}+\sigma^{2}}\right)-\log_{2}\left(1+\frac{P_{k,b}\gamma_{T_{k,b},E_{g}}}{\kappa_{E}^{2}P_{k,b}\gamma_{T_{k,b},E_{g}}+\sigma^{2}}\right)\right]\right). \end{array}$$

With the random-and-forward strategy, the end-to-end secrecy capacity of the proposed protocol is given as in [37]:

(17) $$\begin{array}{c} C_{\mathrm{Sec}}^{e2e}=\min_{k=1,2,\ldots ,K}\left(C_{\mathrm{Sec},k}\right). \end{array}$$

## 3. Performance Analysis

In this section, we analyze the average transmit power of the secondary transmitters and the secrecy performance of the proposed protocol in terms of SOP and PNSC.

### 3.1. Average Transmit Power of the Secondary Transmitters

From (8), the average transmit power of $T_{k}$ can be given by the following formula:

(18) $$\begin{array}{cc} E\left\{P_{k,b}\right\} & =\sum_{v=1}^{W-1}\mathrm{Pr}\left(\frac{v}{W}\leq \min\left(\mu_{1}\varphi_{\mathrm{sum},k},\frac{\mu_{2}}{\gamma_{T_{k,b},\mathrm{PU}}}\right)<\frac{v+1}{W}\right)\frac{v}{W}P_{S}\\ & +\mathrm{Pr}\left(1\leq \min\left(\mu_{1}\varphi_{\mathrm{sum},k},\frac{\mu_{2}}{\gamma_{T_{k,b},\mathrm{PU}}}\right)\right)P_{S}, \end{array}$$

where $E\left\{.\right\}$ is an expectation operator. To calculate $E\left\{P_{k,b}\right\}$, we attempt to find CDF of $\min\left(\mu_{1}\varphi_{\mathrm{sum},k},\mu_{2}/\gamma_{T_{k,b},\mathrm{PU}}\right)$. Setting $Z_{\min,k}=\min\left(\mu_{1}\varphi_{\mathrm{sum},k},\mu_{2}/\gamma_{T_{k,b},\mathrm{PU}}\right)$, the CDF of $Z_{\min,k}$ is obtained by

(19) $$\begin{array}{c} F_{Z_{\min,k}}\left(z\right)=1-\left(1-F_{\varphi_{\mathrm{sum},k}}\left(\frac{z}{\mu_{1}}\right)\right)F_{\gamma_{T_{k,b},\mathrm{PU}}}\left(\frac{\mu_{2}}{z}\right). \end{array}$$

Substituting $F_{\gamma_{T_{k,b},\mathrm{PU}}}\left(x\right)=1-\exp\left(-\lambda_{T_{k},\mathrm{PU}}x\right)$ and (4) into (19) yields

(20) $$\begin{array}{c} F_{Z_{\min,k}}\left(z\right)=1-\left[\sum_{t=0}^{N_{D}-1}\frac{1}{t!}{\left(\lambda_{\mathrm{PB},T_{k}}\frac{z}{\mu_{1}}\right)}^{t}\exp\left(-\lambda_{\mathrm{PB},T_{k}}\frac{z}{\mu_{1}}\right)\right]\left(1-\exp\left(-\frac{\lambda_{T_{k},\mathrm{PU}}\mu_{2}}{z}\right)\right). \end{array}$$

Therefore, the average transmit power of $T_{k}$ is written by

(21) $$\begin{array}{c} E\left\{P_{k,b}\right\}=\sum_{v=1}^{W-1}\left(F_{Z_{\min,k}}\left(\frac{v+1}{W}\right)-F_{Z_{\min,k}}\left(\frac{v}{W}\right)\right)\frac{v}{W}P_{S}+\left(1-F_{Z_{\min,k}}\left(1\right)\right)P_{S}. \end{array}$$

### 3.2. Secrecy Outage Probability (SOP)

The end-to-end SOP of the proposed protocol can be calculated by

(22) $$\begin{array}{cc} \mathrm{SOP} & =\mathrm{Pr}(C_{\mathrm{Sec}}^{e2e}<C_{\mathrm{th}})=\mathrm{Pr}\left(\min_{k=1,2,\ldots ,K}\left(C_{\mathrm{Sec},k}\right)<C_{\mathrm{th}}\right)\\ & =1-\prod_{k=1}^{K}\left(1-\mathrm{Pr}\left(C_{\mathrm{Sec},k}<C_{\mathrm{th}}\right)\right)\\ & =1-\prod_{k=1}^{K}\left(1-\mathrm{SO}P_{k}\right), \end{array}$$

where $C_{\mathrm{th}}\left(C_{\mathrm{th}}>0\right)$ is a predetermined outage threshold, and $\mathrm{SO}P_{k}=\mathrm{Pr}\left(C_{\mathrm{Sec},k}<C_{\mathrm{th}}\right)$ is the secrecy outage probability at the *k*-th hop. Using (8) and (16), we can formulate $\mathrm{SO}P_{k}$ by

(23) $$\begin{array}{c} \mathrm{SO}P_{k}=\sum_{v=1}^{W}\mathrm{Pr}\left(P_{k,b}=L_{v}\right)\times \underset{\mathrm{SO}P_{k}\left(L_{v}\right)}{\underset{︸}{\mathrm{Pr}\left(\frac{1+\frac{L_{v}\gamma_{T_{k,b},T_{k+1,c}}}{\kappa_{D}^{2}L_{v}\gamma_{T_{k,b},T_{k+1,c}}+\sigma^{2}}}{1+\frac{L_{v}\gamma_{T_{k,b},E_{g}}}{\kappa_{E}^{2}L_{v}\gamma_{T_{k,b},E_{g}}+\sigma^{2}}}<\rho \right)}}, \end{array}$$

where $\rho =2^{C_{\mathrm{th}}/\left(1-\varepsilon \right)\tau }$.

It is noted from (23) that the secrecy outage event is only considered in the cases where the transmit power $P_{k,b}$ is higher than zero, i.e., $P_{k,b}=L_{v}$ and v≥1.

For the probability $\mathrm{Pr}\left(P_{k,b}=L_{v}\right)$ in (23), it can be calculated by

(24) $$\begin{array}{c} \mathrm{Pr}\left(P_{k,b}=L_{v}\right)=\left\{\begin{array}{c} F_{Z_{\min,k}}\left(\frac{v+1}{W}\right)-F_{Z_{\min,k}}\left(\frac{v}{W}\right),\;\;\mathrm{if}\;v<W\\ 1-F_{Z_{\min,k}}\left(1\right),\;\;\;\;\;\;\;\;\;\;\;\;\;\;\;\;\;\;\;\;\;\;\;\;\;\;\;\;\;\;\;\;\;\;\;\;\mathrm{if}\;v=W\; \end{array}\right. \end{array}$$

Next, let us consider the SOP conditioned on $P_{k,b}=L_{v}$ as marked in (23); we have

(25) $$\begin{array}{cc} \mathrm{SO}P_{k}\left(L_{v}\right) & =\mathrm{Pr}\left(\frac{L_{v}\gamma_{T_{k,b},T_{k+1,c}}}{\kappa_{D}^{2}L_{v}\gamma_{T_{k,b},T_{k+1,c}}+\sigma^{2}}<\rho -1+\frac{L_{v}\gamma_{T_{k,b},E_{g}}}{\kappa_{E}^{2}L_{v}\gamma_{T_{k,b},E_{g}}+\sigma^{2}}\rho \right)\\ & =\mathrm{Pr}\left(\alpha_{0}\gamma_{T_{k,b},T_{k+1,c}}<\alpha_{1,v}+\alpha_{2}\gamma_{T_{k,b},E_{g}}+\alpha_{3,v}\gamma_{T_{k,b},T_{k+1,c}}\gamma_{T_{k,b},E_{g}}\right), \end{array}$$

where

(26) $$\begin{array}{cc} \alpha_{0} & =1-\left(\rho -1\right)\kappa_{D}^{2},\;\alpha_{1,v}=\frac{\sigma^{2}\left(\rho -1\right)}{L_{v}},\;\alpha_{2}=\left(\rho -1\right)\kappa_{E}^{2}+\rho ,\\ \alpha_{3,v} & =\frac{L_{v}}{\sigma^{2}}\left(\left(\rho -1\right)\kappa_{D}^{2}\kappa_{E}^{2}+\kappa_{D}^{2}\rho -\kappa_{E}^{2}\right). \end{array}$$

We can observe from (25) that if $\alpha_{0}\leq 0$ (or $\kappa_{D}^{2}\geq 1/\left(\rho -1\right)$), $\mathrm{SO}P_{k}\left(L_{v}\right)$ always equals 1 for all values of *k* and *v*.

In the following, we derive the exact expressions of $\mathrm{SO}P_{k}\left(L_{v}\right)$ as given in Lemmas 1–3 below.

**Lemma** **1.** *When $\alpha_{0}>0$ and $\alpha_{3,v}>0$$\left(\;\mathrm{or}\;\;\kappa_{D}^{2}>\kappa_{E}^{2}/\left(\rho +\left(\rho -1\right)\kappa_{E}^{2}\right)\right)$, the exact expression of SOP can be given as*

(27) $$\begin{array}{c} \mathrm{SO}P_{k}\left(L_{v}\right)=1+\sum_{n=1}^{N_{D}^{2}}\sum_{m=0}^{N_{E}-1}{\left(-1\right)}^{n+m}\beta_{0}\int_{0}^{\alpha_{0}}\exp\left(\beta_{1}y\right)\exp\left(-\frac{\beta_{2}}{y}\right)dy. \end{array}$$

**Proof.**  At first, when $\alpha_{0}>0$ and $\alpha_{3,v}>0$, we can rewrite $\mathrm{SO}P_{k}\left(L_{v}\right)$ as

(28) $$\begin{array}{cc} \mathrm{SO}P_{k}\left(L_{v}\right) & =\mathrm{Pr}\left(\left(\alpha_{0}-\alpha_{3,v}\gamma_{T_{k,b},E_{g}}\right)\gamma_{T_{k,b},T_{k+1,c}}<\alpha_{1,v}+\alpha_{2}\gamma_{T_{k,b},E_{g}}\right)\\ & =\mathrm{Pr}\left(\gamma_{T_{k,b},E_{g}}\geq \frac{\alpha_{0}}{\alpha_{3,v}}\right)+\mathrm{Pr}\left(\gamma_{T_{k,b},E_{g}}<\frac{\alpha_{0}}{\alpha_{3,v}},\gamma_{T_{k,b},T_{k+1,c}}<\frac{\alpha_{1,v}+\alpha_{2}\gamma_{T_{k,b},E_{g}}}{\alpha_{0}-\alpha_{3,v}\gamma_{T_{k,b},E_{g}}}\right)\\ & =1-F_{\gamma_{T_{k,b},E_{g}}}\left(\frac{\alpha_{0}}{\alpha_{3,v}}\right)+\int_{0}^{\alpha_{0}/\alpha_{3,v}}F_{\gamma_{T_{k,b},T_{k+1,c}}}\left(\frac{\alpha_{1,v}+\alpha_{2}x}{\alpha_{0}-\alpha_{3,v}x}\right)f_{\gamma_{T_{k,b},E_{g}}}\left(x\right)dx. \end{array}$$

By substituting the CDF of $\gamma_{T_{k,b},T_{k+1,c}}$ in (10), and the PDF of $\gamma_{T_{k,b},E_{g}}$ in (15) into (28), after some manipulations, we arrive at

(29) $$\begin{array}{cc} \mathrm{SO}P_{k}\left(L_{v}\right) & =1+\sum_{n=1}^{N_{D}^{2}}\sum_{m=0}^{N_{E}-1}{\left(-1\right)}^{n+m}\left(\begin{array}{c} N_{D}^{2}\\ n \end{array}\right)\left(\begin{array}{c} N_{E}-1\\ m \end{array}\right)N_{E}\lambda_{T_{k},E}\\ & \times \int_{0}^{\alpha_{0}/\alpha_{3,v}}\exp\left(-\left(m+1\right)\lambda_{T_{k},E}x\right)\exp\left(-n\lambda_{T_{k},T_{k+1}}\frac{\alpha_{1,v}+\alpha_{2}x}{\alpha_{0}-\alpha_{3,v}x}\right)dx. \end{array}$$

By changing variable $y=\alpha_{0}-\alpha_{3,v}x$, we have

(30) $$\begin{array}{cc} \mathrm{SO}P_{k}\left(L_{v}\right)=1+ & \sum_{n=1}^{N_{D}^{2}}\sum_{m=0}^{N_{E}-1}{\left(-1\right)}^{n+m}\left(\begin{array}{c} N_{D}^{2}\\ n \end{array}\right)\left(\begin{array}{c} N_{E}-1\\ m \end{array}\right)\frac{N_{E}\lambda_{T_{k},E}}{\alpha_{3,v}}\\ & \times \exp\left(n\lambda_{T_{k},T_{k+1}}\frac{\alpha_{2}}{\alpha_{3,v}}-\left(m+1\right)\lambda_{T_{k},E}\frac{\alpha_{0}}{\alpha_{3,v}}\right)\\ & \times \int_{0}^{\alpha_{0}}\exp\left(\frac{\left(m+1\right)\lambda_{T_{k},E}}{\alpha_{3,v}}y\right)\exp\left(-n\lambda_{T_{k},T_{k+1}}\frac{\alpha_{1,v}\alpha_{3,v}+\alpha_{0}\alpha_{2}}{\alpha_{3,v}y}\right)dy. \end{array}$$

With $\alpha_{1,v}\alpha_{3,v}+\alpha_{0}\alpha_{2}=\rho$, we can rewrite (30) as

(31) $$\begin{array}{c} \mathrm{SO}P_{k}\left(L_{v}\right)=1+\sum_{n=1}^{N_{D}^{2}}\sum_{m=0}^{N_{E}-1}{\left(-1\right)}^{n+m}\beta_{0}\int_{0}^{\alpha_{0}}\exp\left(\beta_{1}y\right)\exp\left(-\frac{\beta_{2}}{y}\right)dy. \end{array}$$

where

(32) $$\begin{array}{cc} \beta_{0} & =\left(\begin{array}{c} N_{D}^{2}\\ n \end{array}\right)\left(\begin{array}{c} N_{E}-1\\ m \end{array}\right)\frac{N_{E}\lambda_{T_{k},E}}{\alpha_{3,v}}\exp\left(\frac{n\lambda_{T_{k},T_{k+1}}\alpha_{2}-\left(m+1\right)\lambda_{T_{k},E}\alpha_{0}}{\alpha_{3,v}}\right),\\ \beta_{1} & =\frac{\left(m+1\right)\lambda_{T_{k},E}}{\alpha_{3,v}},\beta_{2}=\frac{n\lambda_{T_{k},T_{k+1}}\rho }{\alpha_{3,v}}. \end{array}$$

We finish the proof of Lemma 1 here. We note that the integrals in (27) can be easily calculated by computer software such as Matlab or Mathematica. ☐

**Comment 2**: We can observe from (27) that the exact expression of $\mathrm{SO}P_{k}\left(L_{v}\right)$ is still in integral form, which does not provide any insights into the system performance. Therefore, our next objective is to find an asymptotic expression at high transmit SNR as given in Corollary 1 below.

**Corollary** **1.** *When $\alpha_{0}>0$ and $\alpha_{3,v}>0$, we can approximate $\mathrm{SO}P_{k}\left(L_{v}\right)$ at high transmit SNR $\left(P_{S}/\sigma^{2}\to +\infty \right)$ by a closed-form expression as*

(33) $$\begin{array}{c} \mathrm{SO}P_{k}\left(L_{v}\right)\overset{P_{S}/\sigma^{2}\to +\infty }{\approx }1-{\left(1-\exp\left(-\lambda_{T_{k},E}\frac{\alpha_{0}}{\alpha_{3,v}}\right)\right)}^{N_{E}}. \end{array}$$

**Proof.**  At high transmit SNR, we can approximate (25) in this case as follows:

(34) $$\begin{array}{cc} \mathrm{SO}P_{k}\left(L_{v}\right) & =\mathrm{Pr}\left(\alpha_{0}\gamma_{T_{k,b},T_{k+1,c}}<\alpha_{1,v}+\alpha_{2}\gamma_{T_{k,b},E_{g}}+\alpha_{3,v}\gamma_{T_{k,b},T_{k+1,c}}\gamma_{T_{k,b},E_{g}}\right)\\ & \overset{P_{S}/\sigma^{2}\to +\infty }{\approx }\mathrm{Pr}\left(\alpha_{0}\gamma_{T_{k,b},T_{k+1,c}}<\alpha_{3,v}\gamma_{T_{k,b},T_{k+1,c}}\gamma_{T_{k,b},E_{g}}\right)=\mathrm{Pr}\left(\gamma_{T_{k,b},E_{g}}>\frac{\alpha_{0}}{\alpha_{3,v}}\right)\\ & \overset{P_{S}/\sigma^{2}\to +\infty }{\approx }1-F_{\gamma_{T_{k,b},E_{g}}}\left(\frac{\alpha_{0}}{\alpha_{3,v}}\right). \end{array}$$

Substituting (14) into (34), we obtain (33). ☐

**Comment 3**: Combining (22), (23), (27), and (33), we obtain exact and asymptotic formulas of the end-to-end SOP when $\alpha_{0}>0$ and $\alpha_{3,v}>0$. Equation (33) implies that $\mathrm{SO}P_{k}\left(L_{v}\right)$ at high transmit SNR only depends on $\lambda_{T_{k},E},\;\alpha_{0}$, and $\alpha_{3,v}$. Moreover, $\mathrm{SO}P_{k}\left(L_{v}\right)$ (and the end-to-end SOP) increases when $P_{S}/\sigma^{2}$ increases.

**Lemma** **2.** *When $\alpha_{0}>0$ and $\alpha_{3,v}<0$, an exact expression of $\mathrm{SO}P_{k}\left(L_{v}\right)$ can be given as*

(35) $$\begin{array}{c} \mathrm{SO}P_{k}\left(L_{v}\right)=1+\sum_{n=1}^{N_{D}^{2}}\sum_{m=0}^{N_{E}-1}{\left(-1\right)}^{n+m+1}\beta_{0}\int_{\alpha_{0}}^{+\infty }\exp\left(\beta_{1}y\right)\exp\left(-\frac{\beta_{2}}{y}\right)dy. \end{array}$$

**Proof.**  Similar to (28), $\mathrm{SO}P_{k}\left(L_{v}\right)$ in this case can be written by

(36) $$\begin{array}{cc} \mathrm{SO}P_{k}\left(L_{v}\right) & =\mathrm{Pr}\left(\left(\alpha_{0}-\alpha_{3,v}\gamma_{T_{k,b},E_{g}}\right)\gamma_{T_{k,b},T_{k+1,c}}<\alpha_{1,v}+\alpha_{2}\gamma_{T_{k,b},E_{g}}\right)\\ & =\mathrm{Pr}\left(\gamma_{T_{k,b},T_{k+1,c}}<\frac{\alpha_{1,v}+\alpha_{2}\gamma_{T_{k,b},E_{g}}}{\alpha_{0}-\alpha_{3,v}\gamma_{T_{k,b},E_{g}}}\right)\\ & =\int_{0}^{+\infty }F_{\gamma_{T_{k,b},T_{k+1,c}}}\left(\frac{\alpha_{1,v}+\alpha_{2}x}{\alpha_{0}-\alpha_{3,v}x}\right)f_{\gamma_{T_{k,b},E_{g}}}\left(x\right)dx. \end{array}$$

With the same manner as deriving $\mathrm{SO}P_{k}\left(L_{v}\right)$ in (28), we can obtain (35). ☐

**Corollary** **2.** *At high transmit SNR, $\mathrm{SO}P_{k}\left(L_{v}\right)$ in (35) can be approximated by*

(37) $$\begin{array}{c} \mathrm{SO}P_{k}\left(L_{v}\right)\overset{P_{S}/\sigma^{2}\to +\infty }{\approx }{\left(1-\exp\left(\lambda_{T_{k},T_{k+1}}\frac{\alpha_{2}}{\alpha_{3,v}}\right)\right)}^{N_{D}^{2}}. \end{array}$$

**Proof.**  Similar to the proof of Corollary 1, we have

(38) $$\begin{array}{cc} \mathrm{SO}P_{k}\left(L_{v}\right) & =\mathrm{Pr}\left(\alpha_{0}\gamma_{T_{k,b},T_{k+1,c}}<\alpha_{1,v}+\alpha_{2}\gamma_{T_{k,b},E_{g}}+\alpha_{3,v}\gamma_{T_{k,b},T_{k+1,c}}\gamma_{T_{k,b},E_{g}}\right)\\ & \overset{P_{S}/\sigma^{2}\to +\infty }{\approx }\mathrm{Pr}\left(-\alpha_{3,v}\gamma_{T_{k,b},T_{k+1,c}}\gamma_{T_{k,b},E_{g}}<\alpha_{2}\gamma_{T_{k,b},E_{g}}\right)\\ & \overset{P_{S}/\sigma^{2}\to +\infty }{\approx }F_{\gamma_{T_{k,b},T_{k+1,c}}}\left(-\frac{\alpha_{2}}{\alpha_{3,v}}\right). \end{array}$$

Substituting (10) into (38), we then obtain (37). ☐

**Comment 4**: Combining (22), (23), (35), and (37), we obtain exact and asymptotic expressions of the end-to-end SOP when $\alpha_{0}>0$ and $\alpha_{3,v}<0$. Equation (37) also shows that at high transmit SNR, $\mathrm{SO}P_{k}\left(L_{v}\right)$ (and the end-to-end SOP) decreases as $P_{S}/\sigma^{2}$ increases.

**Lemma** **3.** *When $\alpha_{0}>0$ and $\alpha_{3,v}=0$, $\mathrm{SO}P_{k}\left(L_{v}\right)$ is given by an exact closed-form expression as*

(39) $$\begin{array}{c} \mathrm{SO}P_{k}\left(L_{v}\right)=1+\\ \sum_{n=1}^{N_{D}^{2}}\sum_{m=0}^{N_{E}-1}{\left(-1\right)}^{n+m}\left(\;\;\begin{array}{c} N_{D}^{2}\\ n \end{array}\;\;\right)\left(\;\;\begin{array}{c} N_{E}-1\\ m \end{array}\;\;\right)\frac{N_{E}\lambda_{T_{k},E}}{\left(m+1\right)\lambda_{T_{k},E}+n\lambda_{T_{k},T_{k+1}}\alpha_{2}/\alpha_{0}}\exp\left(-n\lambda_{T_{k},T_{k+1}}\frac{\alpha_{1,v}}{\alpha_{0}}\right). \end{array}$$

**Proof.**  In this case, we have

(40) $$\begin{array}{cc} \mathrm{SO}P_{k}\left(L_{v}\right) & =\mathrm{Pr}\left(\alpha_{0}\gamma_{T_{k,b},T_{k+1,c}}<\alpha_{1,v}+\alpha_{2}\gamma_{T_{k,b},E_{g}}\right)\\ & =\int_{0}^{+\infty }F_{\gamma_{T_{k,b},T_{k+1,c}}}\left(\frac{\alpha_{1,v}}{\alpha_{0}}+\frac{\alpha_{2}}{\alpha_{0}}x\right)f_{\gamma_{T_{k,b},E_{g}}}\left(x\right)dx. \end{array}$$

Substituting CDF of $\gamma_{T_{k,b},T_{k+1,c}}$ in (10), and PDF of $\gamma_{T_{k,b},E_{g}}$ in (15) into (40), after some manipulations, we can obtain (39), and finish the proof. ☐

**Corollary** **3.** *When $\alpha_{0}>0$ and $\alpha_{3}=0$, $\mathrm{SO}P_{k}\left(L_{v}\right)$ can be approximated by*

(41) $$\begin{array}{c} \mathrm{SO}P_{k}\left(L_{v}\right)\overset{P_{S}/\sigma^{2}\to +\infty }{\approx }1+\sum_{n=1}^{N_{D}^{2}}\sum_{m=0}^{N_{E}-1}{\left(-1\right)}^{n+m}\left(\;\;\begin{array}{c} N_{D}^{2}\\ n \end{array}\;\;\right)\left(\;\;\begin{array}{c} N_{E}-1\\ m \end{array}\;\;\right)\frac{N_{E}\lambda_{T_{k},E}}{\left(m+1\right)\lambda_{T_{k},E}+n\lambda_{T_{k},T_{k+1}}\alpha_{2}/\alpha_{0}}. \end{array}$$

**Proof.**  In this case, we have the following approximation:

(42) $$\begin{array}{cc} \mathrm{SO}P_{k}\left(L_{v}\right) & =\mathrm{Pr}\left(\alpha_{0}\gamma_{T_{k,b},T_{k+1,c}}<\alpha_{1,v}+\alpha_{2}\gamma_{T_{k,b},E_{g}}\right)\\ & \overset{P_{S}/\sigma^{2}\to +\infty }{\approx }\mathrm{Pr}\left(\alpha_{0}\gamma_{T_{k,b},T_{k+1,c}}<\alpha_{2}\gamma_{T_{k,b},E_{g}}\right)\\ & \overset{P_{S}/\sigma^{2}\to +\infty }{\approx }\int_{0}^{+\infty }F_{\gamma_{T_{k,b},T_{k+1,c}}}\left(\frac{\alpha_{2}}{\alpha_{0}}x\right)f_{\gamma_{T_{k,b},E_{g}}}\left(x\right)dx. \end{array}$$

 ☐

Substituting CDF of $\gamma_{T_{k,b},T_{k+1,c}}$, and PDF of $\gamma_{T_{k,b},E_{g}}$ into (42), after some manipulations, we can obtain (41).

**Comment 5**: Equation (41) shows that $\mathrm{SO}P_{k}\left(L_{v}\right)$, as well as the end-to-end SOP at high transmit SNR, do not depend on $P_{S}/\sigma^{2}$. It is worth noting that this paper considers a generalized system model where the hardware impairment levels on the data links and eavesdropping links can be different or the same. Moreover, we can observe from Lemmas 1–3 that the secrecy outage probability of the proposed protocol is only expressed by an exact closed-form formula when $\alpha_{3}=0$.

### 3.3. Probability of Non-Zero Secrecy Capacity (PNSC)

In this subsection, we analyze the end-to-end PNSC of the proposed protocol, which can be formulated by

(43) $$\begin{array}{cc} \mathrm{PNSC} & =\mathrm{Pr}(C_{\mathrm{Sec}}^{e2e}>0)=\mathrm{Pr}\left(\min_{k=1,2,\ldots ,K}\left(C_{\mathrm{Sec},k}\right)>0\right)\\ & =\prod_{k=1}^{K}\mathrm{Pr}\left(C_{\mathrm{Sec},k}>0\right)\\ & =\prod_{k=1}^{K}\mathrm{PNS}C_{k}, \end{array}$$

where $\mathrm{PNS}C_{k}=\mathrm{Pr}\left(C_{\mathrm{Sec},k}>0\right)$ is the probability of non-zero secrecy capacity at the *k*-th hop.

**Lemma** **4.** *The exact expressions of $\mathrm{PNS}C_{k}$ can be given by*

(44) $$\begin{array}{cc} \mathrm{PNS}C_{k} & =\sum_{v=1}^{M}\mathrm{Pr}\left(P_{k,b}=L_{v}\right)\\ & \times \left\{\begin{array}{c} \sum_{n=1}^{N_{D}^{2}}\sum_{m=0}^{N_{E}-1}{\left(-1\right)}^{n+m+1}\chi_{0}\int_{0}^{1}\exp\left(\chi_{1}y\right)\exp\left(-\frac{\chi_{2}}{y}\right)dy,\;\;\;\;\;\;\;\;\;\;\;\mathrm{if}\;\;\kappa_{D}^{2}>\kappa_{E}^{2}\\ \sum_{n=1}^{N_{D}^{2}}\sum_{m=0}^{N_{E}-1}{\left(-1\right)}^{n+m+1}\chi_{0}\int_{1}^{+\infty }\exp\left(\chi_{1}y\right)\exp\left(-\frac{\chi_{2}}{y}\right)dy,\;\;\;\;\;\;\;\mathrm{if}\;\;\kappa_{D}^{2}<\kappa_{E}^{2}\\ \sum_{n=1}^{N_{D}^{2}}\sum_{m=0}^{N-1}{\left(-1\right)}^{n+m}\left(\begin{array}{c} N_{D}^{2}\\ n \end{array}\right)\left(\begin{array}{c} N_{E}-1\\ m \end{array}\right)\frac{N_{E}\lambda_{T_{k},E}}{\left(m+1\right)\lambda_{T_{k},E}+n\lambda_{T_{k},T_{k+1}}},\;\mathrm{if}\;\;\kappa_{D}^{2}=\kappa_{E}^{2} \end{array}\right. \end{array}$$

*where $\mathrm{Pr}\left(P_{k,b}=L_{v}\right)$ is calculated as in (24), and*

(45) $$\begin{array}{cc} \chi_{0} & =\left(\begin{array}{c} N_{D}^{2}\\ n \end{array}\right)\left(\begin{array}{c} N_{E}-1\\ m \end{array}\right)\frac{N_{E}\lambda_{T_{k},E}\sigma^{2}}{L_{v}\left(\kappa_{D}^{2}-\kappa_{E}^{2}\right)}\exp\left(\frac{\left(n\lambda_{T_{k},T_{k+1}}-\left(m+1\right)\lambda_{T_{k},E}\right)\sigma^{2}}{L_{v}\left(\kappa_{D}^{2}-\kappa_{E}^{2}\right)}\right),\\ \chi_{1} & =\frac{\left(m+1\right)\lambda_{T_{k},E}\sigma^{2}}{L_{v}\left(\kappa_{D}^{2}-\kappa_{E}^{2}\right)},\chi_{2}=\frac{n\lambda_{T_{k},T_{k+1}}\sigma^{2}}{L_{v}\left(\kappa_{D}^{2}-\kappa_{E}^{2}\right)}. \end{array}$$

**Proof.**  Similar to (23), we can formulate $\mathrm{PNS}C_{k}$ as

(46) $$\begin{array}{cc} \mathrm{PNS}C_{k} & =\sum_{v=1}^{M}\mathrm{Pr}\left(P_{k,b}=L_{v}\right)\times \mathrm{Pr}\left(\frac{1+\frac{L_{v}\gamma_{T_{k,b},T_{k+1,c}}}{\kappa_{D}^{2}L_{v}\gamma_{T_{k,b},T_{k+1,c}}+\sigma^{2}}}{1+\frac{L_{v}\gamma_{T_{k,b},E_{g}}}{\kappa_{E}^{2}L_{v}\gamma_{T_{k,b},E_{g}}+\sigma^{2}}}>1\right)\\ & =\sum_{v=1}^{M}\mathrm{Pr}\left(P_{k,b}=L_{v}\right)\times \left[1-\lim_{\rho \to 1}\left(\mathrm{SO}P_{k}\left(L_{v}\right)\right)\right]. \end{array}$$

Next, by substituting ρ=1, $\alpha_{0}=1,\;\;\alpha_{1,v}=0,\;\;\alpha_{2}=1$ into $\alpha_{3,v}=L_{v}\left(\kappa_{D}^{2}-\kappa_{E}^{2}\right)/\sigma^{2}$ in (27), (35), and (39), we can obtain (45). ☐

**Comment 6**: Similar to [37], the end-to-end PNSC can be obtained with three different cases, i.e., $\kappa_{D}^{2}>\kappa_{E}^{2}$, $\kappa_{D}^{2}<\kappa_{E}^{2}$, and $\kappa_{D}^{2}=\kappa_{E}^{2}$. Moreover, when $\kappa_{D}^{2}=\kappa_{E}^{2}$, we obtain the exact closed-form expression of the end-to-end PNSC. Finally, with $\kappa_{D}^{2}=\kappa_{E}^{2}$, the end-to-end PNSC value does not depend on the transmit SNR as well as the hardware impairment levels.

## 4. Simulation Results

In this section, we present Monte Carlo simulations to verify the theoretical results obtained in Section 3 by using MATLAB 2014a. For Monte Carlo experiments, we perform $10^{5}$–$5*10^{6}$ independent trials, and in each trial, the Rayleigh channel coefficients for all of the links are generated to obtain the end-to-end secrecy performance. For the theoretical results, the expressions derived in Section 3 are used to present them.

In the simulation environment, a two-dimensional Oxy plane is considered, where the primary user (PU), the power beacon (PB), the secondary eavesdropper (E), and the secondary node ($T_{k}$) are located at $\left(x_{P},y_{P}\right)$, $\left(x_{B},y_{B}\right)$, $\left(x_{E},y_{E}\right)$, and $\left(k/K,\;0\right)$, respectively, where k=0,1,…,K. In all of the simulations, we fix the path-loss exponent $\left(\beta \right)$ by 3, the number of transmit power levels $\left(W\right)$ by 8, the variance of Gaussian noises $\left(\sigma^{2}\right)$ by 1, and the block time (T) by 1. Moreover, we assume that $P_{B}=2P_{S}=4I_{\mathrm{th}}$, and the total hardware impairment level $\kappa_{P}^{2}$ equals 0. It is noted from figures that simulation results (Sim) are presented by markers, while the theoretical results including exact ones (Exact) and asymptotic ones (Asym) are presented by solid and dash lines, respectively.

**Figure 2:**
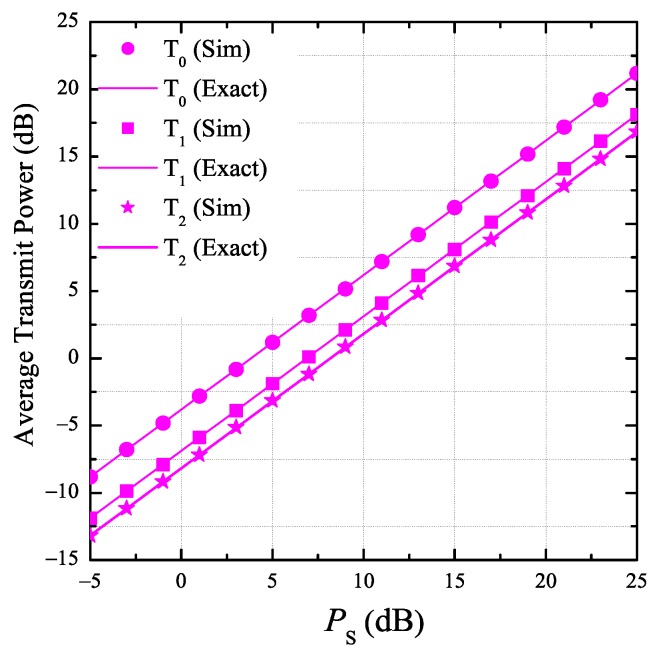
Average transmit power of the secondary transmitters as a function of $P_{S}$ when K=3, $N_{D}=3$, ε=0.25, η=0.25, $x_{B}=0.4$, $y_{B}=0.3$, $x_{P}=0.6$, and $y_{P}=-0.5$.

In Figure 2, we present the average transmit power of the secondary transmitters as a function of $P_{S}$. In this simulation, the number of hops (*K*) is 3, the number of antennas at the node $T_{k}$$\left(N_{D}\right)$ equals 3, the energy conversion efficiency $\left(\eta \right)$ by 0.25, and the fraction of time spent for the EH phase $\left(\varepsilon \right)$ is 0.25. In addition, the co-ordinates of PB and PU are $\left(0.4,\;0.3\right)$ and $\left(0.6,\;-0.5\right)$, respectively. We can observe from Figure 2 that the average transmit power of the source ($T_{0}$) is highest because the Euclidean distance between $T_{0}$ and PU is farthest. We also see that the average transmit power of $T_{0}$, $T_{1}$, and $T_{2}$ linearly increases as $P_{S}$ increases. It is worth noting that the simulation results match very well with the theoretical ones, which validates our derivations.

**Figure 3:**
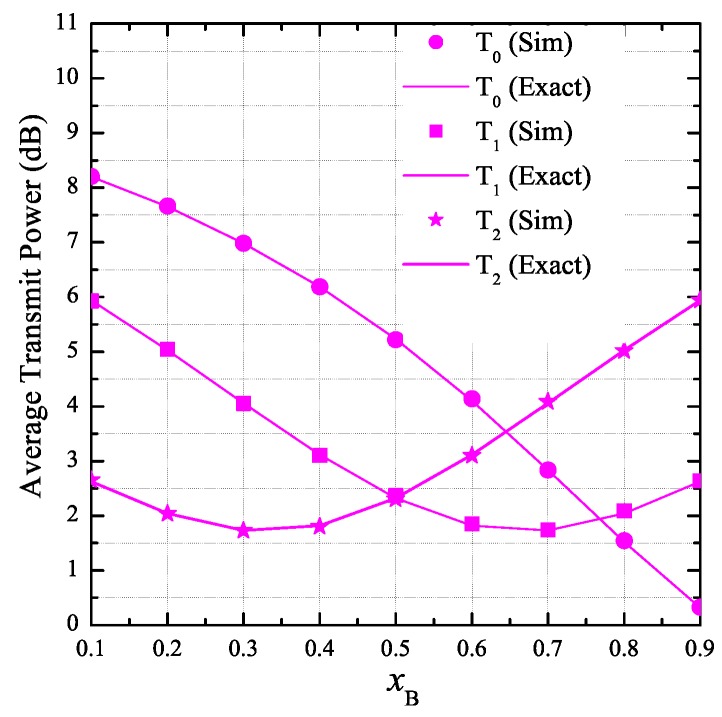
Average transmit power of the secondary transmitters as a function of $x_{B}$ when $P_{S}=10$ dB, K=3, $N_{D}=3$, ε=0.25, η=0.25, $y_{B}=0.3$, $x_{P}=1-x_{B}$, and $y_{P}=-0.5$.

Figure 3 investigates the impact of the positions of PU and PB on the average transmit power of the secondary transmitters. Particularly, we change $x_{B}$ from 0.1 to 0.9, and $x_{P}$ is calculated by $x_{P}=1-x_{B}$. Moreover, we fix the values of $y_{B}$ and $y_{P}$ by 0.3 and –0.5, respectively. It can be seen from Figure 3 that the average transmit power of $T_{0}$, $T_{1}$, and $T_{2}$ varies with different positions of PB and PU. For example, when $x_{B}=0.1$, the positions of PB and PU are (0.1, 0.3) and (0.9, –0.5), respectively. In this case, the average transmit power of $T_{2}$ is lowest because this node is nearest to PU. For another example, $x_{B}=0.5$, the distances between $T_{1}$ and PU, and between $T_{2}$ and PU are the same, and hence the average transmit power of $T_{1}$ and $T_{2}$ is almost the same. For the nodes $T_{1}$ and $T_{2}$, we see that there exist positions of PB and PU at which their average transmit power is lowest. However, the average transmit power of $T_{0}$ always decreases as $x_{B}$ increases.

**Figure 4:**
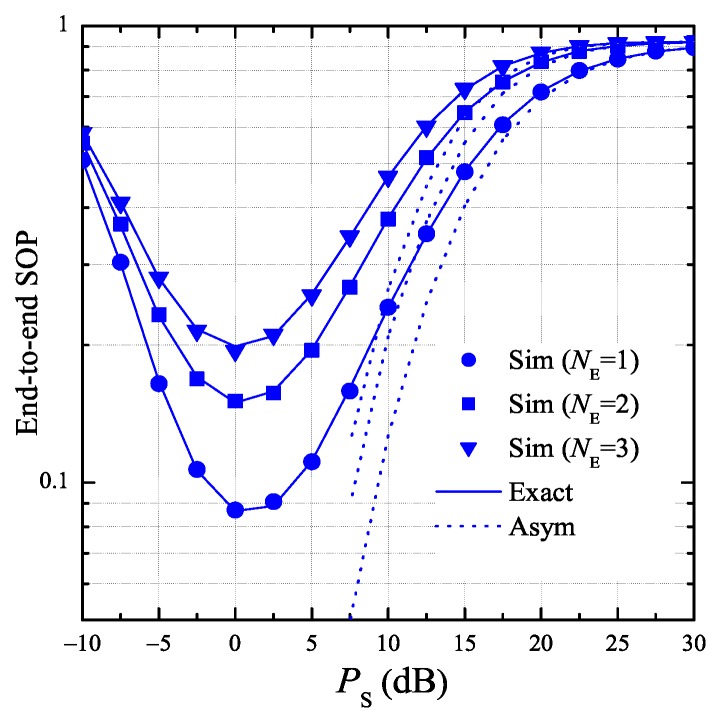
End-to-end secrecy outage probability as a function of $P_{S}$ when K=3, $N_{D}=2$, ε=0.25, η=0.25, $C_{\mathrm{th}}=0.2$, $x_{B}=0.5$, $y_{B}=0.3$, $x_{P}=0.5$, $y_{P}=-0.5$, $x_{E}=0.5$, $y_{E}=0.5$, $\kappa_{D}^{2}=0.01$, and $\kappa_{E}^{2}=0$.

Figure 4 presents the end-to-end SOP as a function of $P_{S}$ with different number of antennas at E. In this simulation, the total hardware impairment levels of the data and eavesdropping links are given as $\kappa_{D}^{2}=0.01$ and $\kappa_{E}^{2}=0$, respectively, and hence the conditions in Lemma 1 are satisfied, i.e., $\alpha_{0}>0$ and $\alpha_{3,v}>0$. It can be seen from this figure that simulation results match very well with theoretical ones. Moreover, we see that the exact end-to-end SOP converges to the approximate one at high $P_{S}$ values, which verifies the derived expressions obtained in Lemma 1 and Corollary 1. As proved in Corollary 1, the end-to-end SOP at high $P_{S}$ regime increases with the increasing of $P_{S}$. In addition, Figure 4 shows that there exists an optimal value of $P_{S}$ at which the SOP value is lowest. Finally, we can see that the secrecy performance of the proposed protocol is worse with higher number of antennas at E.

**Figure 5:**
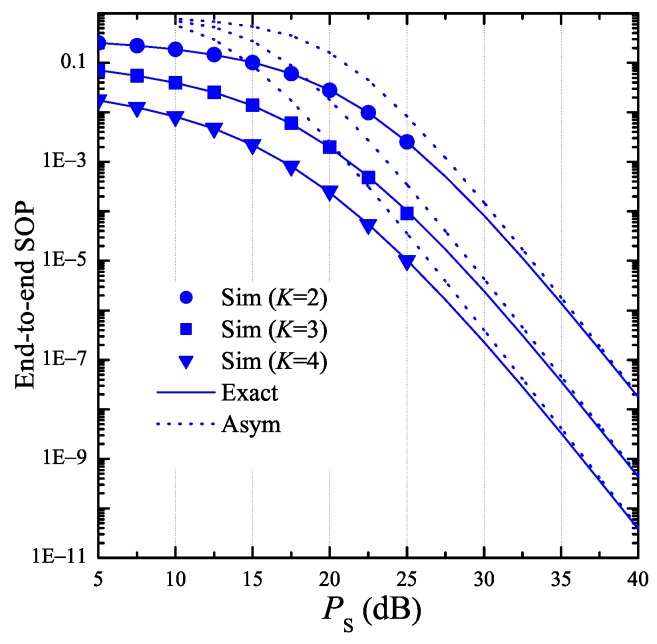
End-to-end secrecy outage probability as a function of $P_{S}$ when $N_{D}=2$, $N_{E}=2$, ε=0.25, η=0.25, $C_{\mathrm{th}}=0.2$, $x_{B}=0.5$, $y_{B}=0.3$, $x_{P}=0.5$, $y_{P}=-0.5$, $x_{E}=0.5$, $y_{E}=0.5$, $\kappa_{D}^{2}=0$, and $\kappa_{E}^{2}=0.01$.

In Figure 5, we present the end-to-end SOP as a function of $P_{S}$ with various number of hops $\left(K\right)$. In this figure, we assume that the transceiver hardware of the authorized nodes is better than that of the eavesdropper, i.e., $\kappa_{D}^{2}=0$ and $\kappa_{E}^{2}=0.01$, which satisfy the conditions in Lemma 2, i.e., $\alpha_{0}>0$ and $\alpha_{3,v}<0$. It is seen from Figure 5 that the end-to-end SOP rapidly decreases as $P_{S}$ increases. Moreover, the secrecy performance significantly enhances with higher number of hops. Finally, the simulation results again validate the theoretical results obtained from Lemma 2 and Corollary 2.

**Figure 6:**
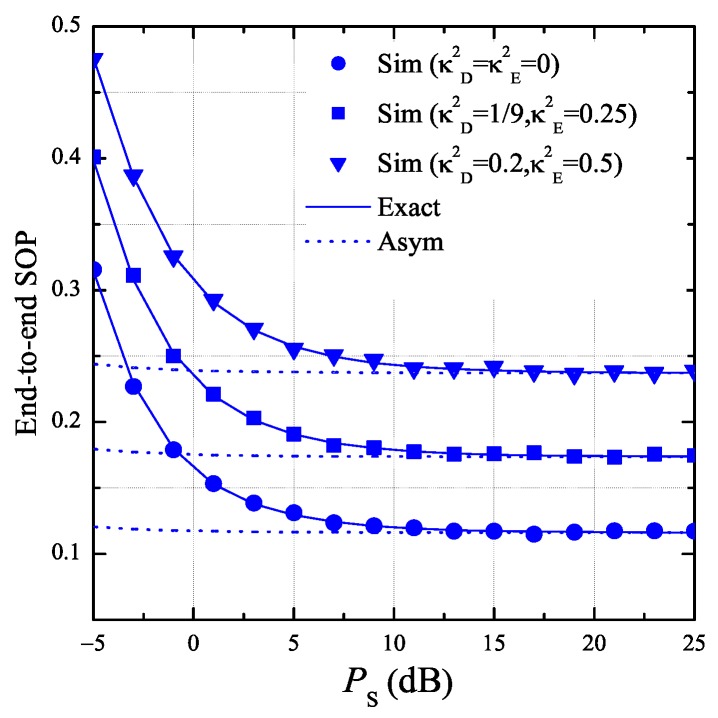
End-to-end secrecy outage probability as a function of $P_{S}$ when K=3, $N_{D}=2$, $N_{E}=2$, ε=0.25, η=0.25, $C_{\mathrm{th}}=0.25$, $x_{B}=0.5$, $y_{B}=0.3$, $x_{P}=0.5$, $y_{P}=-0.5$, $x_{E}=0.5$, and $y_{E}=0.5$.

In Figure 6, we consider the cases where $\alpha_{3,v}=0$ or $\left(\rho -1\right)\kappa_{D}^{2}\kappa_{E}^{2}+\kappa_{D}^{2}\rho -\kappa_{E}^{2}=0$. As proved in Lemma 3 and Corollary 3, we can see from Figure 6 that the exact end-to-end SOP rapidly converges to the asymptotic one which does not depend on $P_{S}$. It is also seen that the hardware impairment levels $\kappa_{D}^{2}$ and $\kappa_{E}^{2}$ significantly impact on the secrecy performance. In this figure, the value of SOP is lowest when the transceiver hardware of the authorized nodes and the eavesdropper is perfect, i.e., $\kappa_{D}^{2}=\kappa_{E}^{2}=0$.

**Figure 7:**
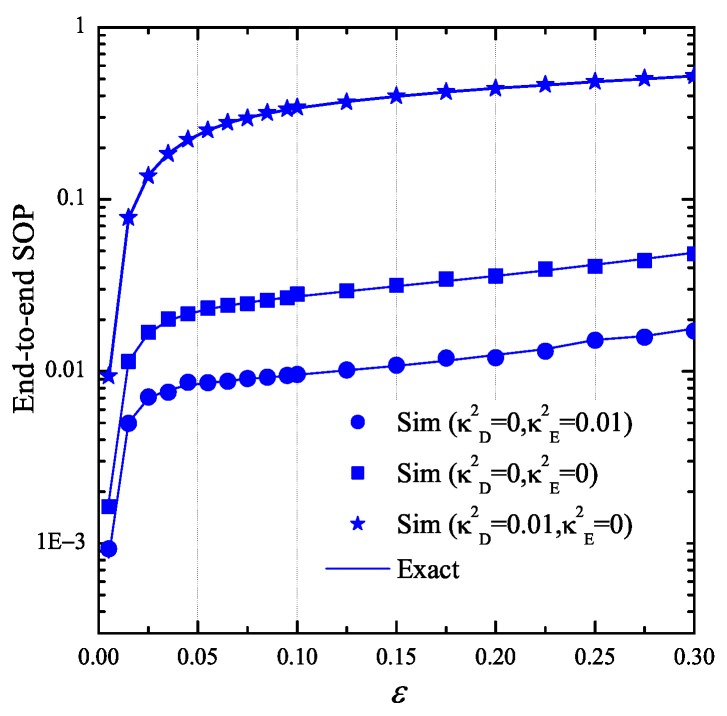
End-to-end secrecy outage probability as a function of ε when $P_{S}=10$ dB, K=4, $N_{D}=2$, $N_{E}=2$, η=0.1, $C_{\mathrm{th}}=0.25$, $x_{B}=0.5$, $y_{B}=0.3$, $x_{P}=0.5$, $y_{P}=-0.5$, $x_{E}=0.5$, and $y_{E}=0.5$.

Figure 7 presents the SOP performance of the proposed protocol as a function of the fraction of time spent for the EH process $\left(\varepsilon \right)$. As illustrated in this figure, the end-to-end SOP increases with higher value of ε. Moreover, when ε is very small, the probability $\mathrm{Pr}\left(P_{k,b}=L_{v}\right)$ in (23) (v≥1) converges to zero, and hence the end-to-end SOP also goes to zero. Next, similar to Figure 5, the secrecy performance significantly enhances when the hardware impairment level of the data links $\left(\kappa_{D}^{2}\right)$ is lower than that of the eavesdropping links $\left(\kappa_{E}^{2}\right)$.

**Figure 8:**
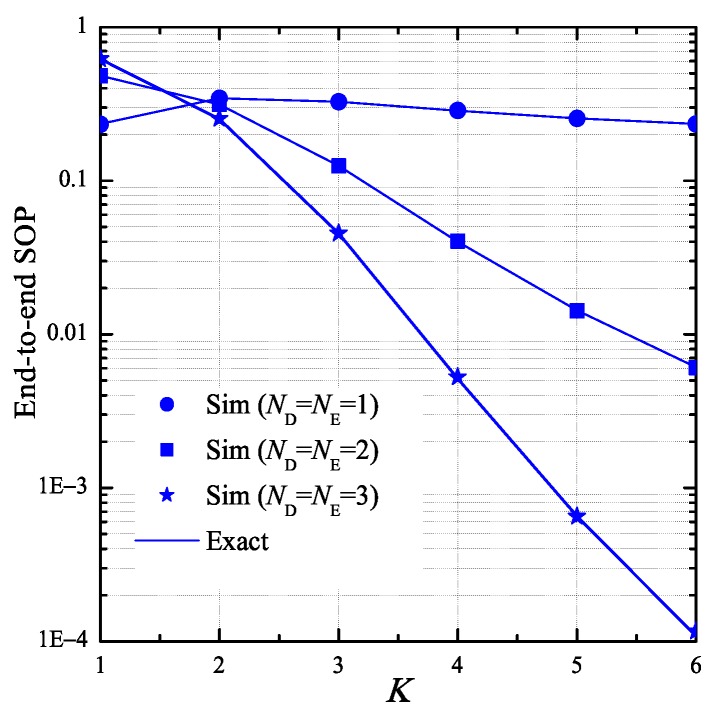
End-to-end secrecy outage probability as a function of *K* when $P_{S}=0$ dB, ε=0.1, η=0.1, $C_{\mathrm{th}}=0.25$, $x_{B}=0.5$, $y_{B}=0.3$, $x_{P}=0.5$, $y_{P}=-0.5$, $x_{E}=0.5$, $y_{E}=0.5$, $\kappa_{D}^{2}=0$, and $\kappa_{E}^{2}=0$.

In Figure 8, we investigate the impact of the number of hops $\left(K\right)$ on the end-to-end SOP. In this simulation, we assume that the number of antennas at the $T_{k}$ and E nodes is same, i.e., $N_{D}=N_{E}$, and the transceiver hardware of these nodes is perfect, i.e., $\kappa_{D}^{2}=\kappa_{E}^{2}=0$. As we can see, the values of SOP almost decrease as increasing the number of hops. However, in the case that $N_{D}=N_{E}=1$, the end-to-end SOP is highest when the number of hops equals 2. When K≥2, it is seen that the SOP performance in case that $N_{D}=N_{E}=3$ is best. Moreover, the value of SOP in this case rapidly decreases with the increasing of *K*.

**Figure 9:**
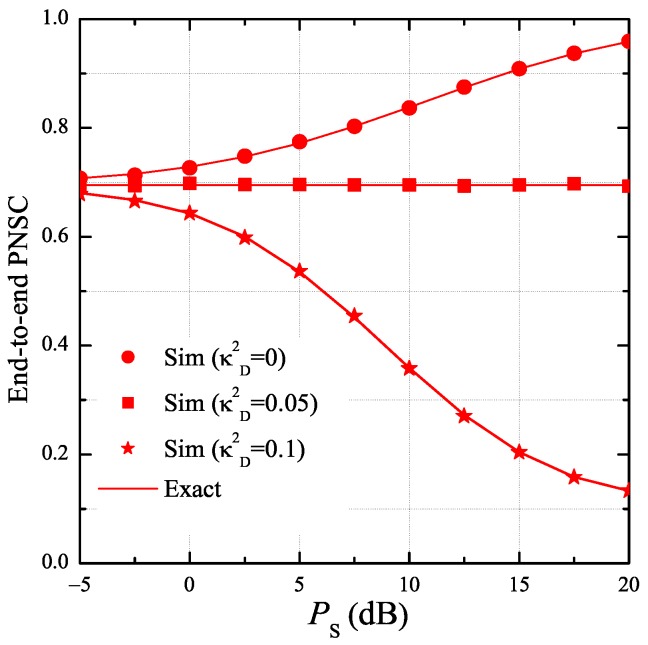
End-to-end probability of non-zero secrecy capacity as a function of $P_{S}$ when K=3, ε=0.25, η=0.25, $x_{B}=0.5$, $y_{B}=0.3$, $x_{P}=0.5$, $y_{P}=-0.5$, $x_{E}=0.5$, $y_{E}=0.5$, $\kappa_{E}^{2}=0.05$, $N_{D}=1$, and $N_{E}=2$.

In Figure 9, we present the end-to-end PNSC as a function of $P_{S}$ with various values of $\kappa_{D}^{2}$ as $\kappa_{E}^{2}$ is fixed by 0.05. As observed, the PNSC performance is better with the decreasing of $\kappa_{D}^{2}$. Moreover, as $\kappa_{D}^{2}<\kappa_{E}^{2}$$\left(\kappa_{D}^{2}>\kappa_{E}^{2}\right)$, the value of PNSC increases (decreases) with higher value of $P_{S}$, and in the case that $\kappa_{D}^{2}=\kappa_{E}^{2}$, this value does not depend on $P_{S}$. It is worth noting that the simulation and theoretical results match very well with each other, which validates the formulas derived in Lemma 4.

**Figure 10:**
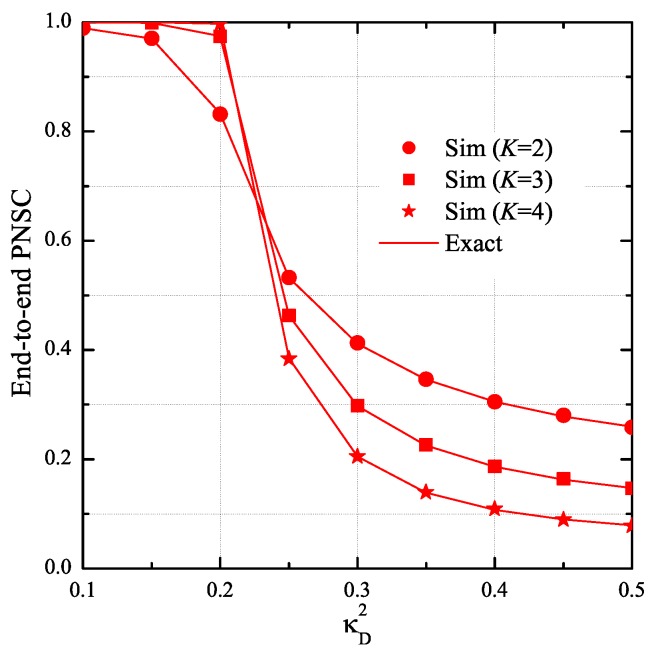
End-to-end probability of non-zero secrecy capacity as a function of $\kappa_{D}^{2}$ when K=3, ε=0.1, η=0.25, $x_{B}=0.5$, $y_{B}=0.3$, $x_{P}=0.5$, $y_{P}=-0.5$, $x_{E}=0.5$, $y_{E}=0.5$, $\kappa_{E}^{2}=0.2$, $N_{D}=2$, and $N_{E}=2$.

Figure 10 presents the end-to-end PNSC as a function of $\kappa_{D}^{2}$ with different number of hops (*K*). As shown in this figure, the PNSC performance significantly decreases when the hardware impairment level of the data link increases. Moreover, at high $\kappa_{D}^{2}$ values, the end-to-end PNSC is worse with high number of hops.

## 5. Conclusions

This paper proposed and evaluated the secrecy performance of the TAS/SC-based multi-hop harvest-to-transmit cognitive WSNs under the joint impact of the interference constraint, the limited-energy source, and the hardware impairments. The main contribution of this paper is to derive new exact and asymptotic expressions of the end-to-end SOP and PNSC over Rayleigh fading channel, which can be used to design and optimize the performance of the considered networks, with any hardware impairment levels, as well as other system parameters, in the practical considerations. The interesting results obtained in this paper can be listed as follows:

- The hardware impairments have a significant impact on the secrecy performance. Particularly, when the transceiver hardware of the authorized nodes is better than that of the eavesdropper, the proposed protocol obtains high secrecy performance. Otherwise, the SOP and PNSC performance is significantly degraded.
- The secrecy performance of the proposed protocol can be enhanced with higher number of antennas equipped at the authorized nodes.
- By optimally designing the number of hops and the fraction of time spent for the energy harvesting phase, the secrecy performance of the proposed protocol can be significantly improved.
